# Efficacy of ibuprofen and indomethacin as prophylaxis of heterotopic ossification: a comparative study

**DOI:** 10.1038/s41598-023-47508-8

**Published:** 2023-11-18

**Authors:** Jens Schneider, Nicola Maffulli, Jörg Eschweiler, Andreas Bell, Frank Hildebrand, Filippo Migliorini

**Affiliations:** 1 Department of Orthopedics and Trauma Surgery, University Clinic Aachen, RWTH Aachen University Clinic, 52064 Aachen, Germany; 2 Department of Orthopedics and Trauma Surgery, Eifelklinik St. Brigida, 52152 Simmerath, Germany; 3 Department of Medicine and Psychology, University of Rome “La Sapienza”, Rome, Italy; 4 Faculty of Medicine, School of Pharmacy and Bioengineering, Keele University, Stoke on Trent, ST4 7QB UK; 5 Barts and the London School of Medicine and Dentistry, Centre for Sports and Exercise Medicine, Mile End Hospital, Queen Mary University of London, London, E1 4DG UK; 6 Department of Orthopaedic and Trauma Surgery, Academic Hospital of Bolzano (SABES-ASDAA), Teaching Hospital of Paracelsus Medical University, 39100 Bolzano, Italy

**Keywords:** Drug discovery, Medical research, Risk factors

## Abstract

The prophylactic action of non-steroidal anti-inflammatory drugs (NSAIDs) in heterotopic ossification (HO) was first described following analgesic therapy with indomethacin. Following that evidence, several compounds have been successfully used for prophylaxes of HO. Ibuprofen has been also proposed for the prevention of HO following THA. The present study compared the administration of ibuprofen for three weeks versus indomethacin as prophylaxis for HO following primary THA. In all THA procedures, pre- and post-operative protocols were conducted in a highly standardized fashion. The type of HO prophylaxis (indomethacin 100 mg/daily or ibuprofen 100 mg/daily) was chosen according to a chronological criterion: from 2017 to 2019 indomethacin was used, whereas from 2019 to 2022 ibuprofen was administered. In case of allergy or intolerance to NSAIDs, no prophylaxis was performed, and patients were included as a control group. All patients who underwent an anteroposterior radiography of the pelvis at a minimum of 12 months following THA were considered for inclusion. On admission, the age and sex of the patients were recorded. Moreover, the causes of osteoarthritis and the date of surgery were recorded. The grade of HO was assigned by a blinded assessor who was not involved in the clinical management of the patients. The modified Brooker Staging System was used to rate the efficacy of the interventions. Data from 1248 patients were collected. 62% (767 of 1248 patients) were women. The mean age was 67.0 ± 2.9 years. The mean follow-up was 21.1 ± 10.8 months. In the ibuprofen group, 73% of patients evidenced Brooker 0, 17% Brooker I, and 10% Brooker II. In the indomethacin group, 77% of patients evidenced Brooker 0, 16% Brooker I, 6% Brooker II. No patient in the ibuprofen and indomethacin group developed Brooker III or IV. In the control group, 64% of patients evidenced Brooker 0, 21% Brooker I, 3% Brooker II, and 12% Brooker III. No patient in the control group developed Brooker IV HO. Concluding, three weeks of administration of ibuprofen demonstrated similar efficacy to indomethacin in preventing HO following primary THA. The prophylaxis with ibuprofen or indomethacin was more effective in preventing HO compared to a control group who did not receive any pharmacological prophylaxis.

## Introduction

Total hip arthroplasty (THA) is considered a milestone in the treatment of end-stage degenerative joint osteoarthritis (OA)^1–3^. Following primary THA, up to 60% of patients develop heterotopic ossification (HO)^4–6^. Advanced HO impairs hip function and is associated with chronic pain, reducing the quality of life of affected patients^7–9^. The aetiology of HO is not fully clarified, and its prophylaxis is indicated in patients following THA^10–16^. Several strategies to prevent HO are available, including prophylactic radiation therapy and non-steroidal anti-inflammatory drugs (NSAIDs)^17–21^. Prophylactic radiation therapy is recommended only for patients at high risk, including bilateral hypertrophic osteoarthritis, prior history of HO, as prophylactic radiation therapy impairs bone and soft tissue healing^22–25^. Prophylactic radiation therapy may prevent acetabulum or proximal femur bone ingrowth, leading to implant failure^23^. Though administered at low doses and using genital shielding, radiation may impair fertility and, rarely, secondary malignancies may develop^23,26,27^. NSAIDs are commonly employed as prophylaxes for HO following THA^10,16,20,28–33^. Though the administration of NSAIDs for HO prevention is off-label in most countries, their analgetic and anti-inflammatory effects justify their use in THA^16,25,33,34^. The prophylactic action of NSAIDs in HO was first described following analgesia with indomethacin^35^. Following that evidence, several compounds have been successfully used for prophylaxes of HO. Ibuprofen has been also proposed for the prevention of HO following THA^36–39^. These compounds are the most commonly used prophylaxis for THA; however, whether ibuprofen is associated with a lower occurrence of HO than indomethacin is unclear. Therefore, the present study was conducted to compare the administration of ibuprofen versus indomethacin as prophylaxis for HO following primary THA.

## Material and methods

### Study design

The present study was performed according to the Strengthening the Reporting of Observational Studies in Epidemiology (STROBE)^40^. The databases of the Department of Orthopaedic Surgery of the Eifelklinik St. Brigida, Simmerath, Germany and University Hospital RWTH Aachen, Germany, were accessed. The records of patients who underwent THA in the period between 2016 and 2022 were accessed for inclusion. All patients who underwent three weeks of HO prophylaxis using ibuprofen 600 mg twice daily or indomethacin 50 mg twice daily were included in the treatment group. Proton pump inhibitors (PPIs) were administered in all patients who underwent HO prophylaxis (pantoprazole 20 mg once daily). Before discharge, patients were educated on the importance of undergoing HO prophylaxis for the entire duration of the therapy as indicated in our postoperative protocol. Patients who did not undergo any prophylaxis for HO were included in the control group. The present study was approved and registered by the ethics committee of the RWTH University of Aachen (project ID: EK128/19), and conducted according to the principles expressed in the Declaration of Helsinki.

### Eligibility criteria

The inclusion criteria were: (1) symptomatic OA secondary to dysplasia, or femoral head necrosis, and idiopathic OA; (2) OA grade II to IV according to the Kellgren-Lawrence classification^41^; (3) only patients who completed the three full weeks of NSAIDs prophylaxis; (4) patients being able to understand the nature of the treatment. The exclusion criteria were: (1) patients who combined other NSAIDs to the HO prophylaxis; (2) OA secondary to trauma; (3) chronic or acute inflammatory diseases; (4) neoplastic diseases; (5) pregnancy; (6) any blood abnormalities; (7) immunodeficiency; (8) severe peripheral neuropathy, vascular diseases, or presence of peripheral ulcers; (9) osteoporosis or other bone ailments which require stem and/or cup cementation; (10) concomitant intake of anticoagulants or calcitonin; (11) other omitted criteria which may have influenced the results of the present investigation.

### Allocation

All the THA procedures, pre- and post-operative protocols were conducted in a highly standardized fashion. The type of HO prophylaxis (indomethacin or ibuprofen) was chosen according to a chronological criterion: from 2017 to 2019, 50 mg twice daily of indomethacin was used. For internal reasons, in 2019 the protocol of HO at our institution changed, and 600 mg twice daily of ibuprofen was introduced. In case of allergy or intolerance to NSAIDs, no prophylaxis was performed and patients were included as a control group. Patients who developed an adverse reaction requiring suspension of the drug, or those who developed a pathology incompatible with the intake of ibuprofen or indomethacin within the first five postoperative days, were invited to discontinue prophylaxis and were included in the control group. In case of suspension of an NSAID, therapy with paracetamol, opioids, or metamizole was started. Patients who discontinued the prophylaxis after discharge were excluded from the present study.

### Outcomes of interest

**Figure 1:**
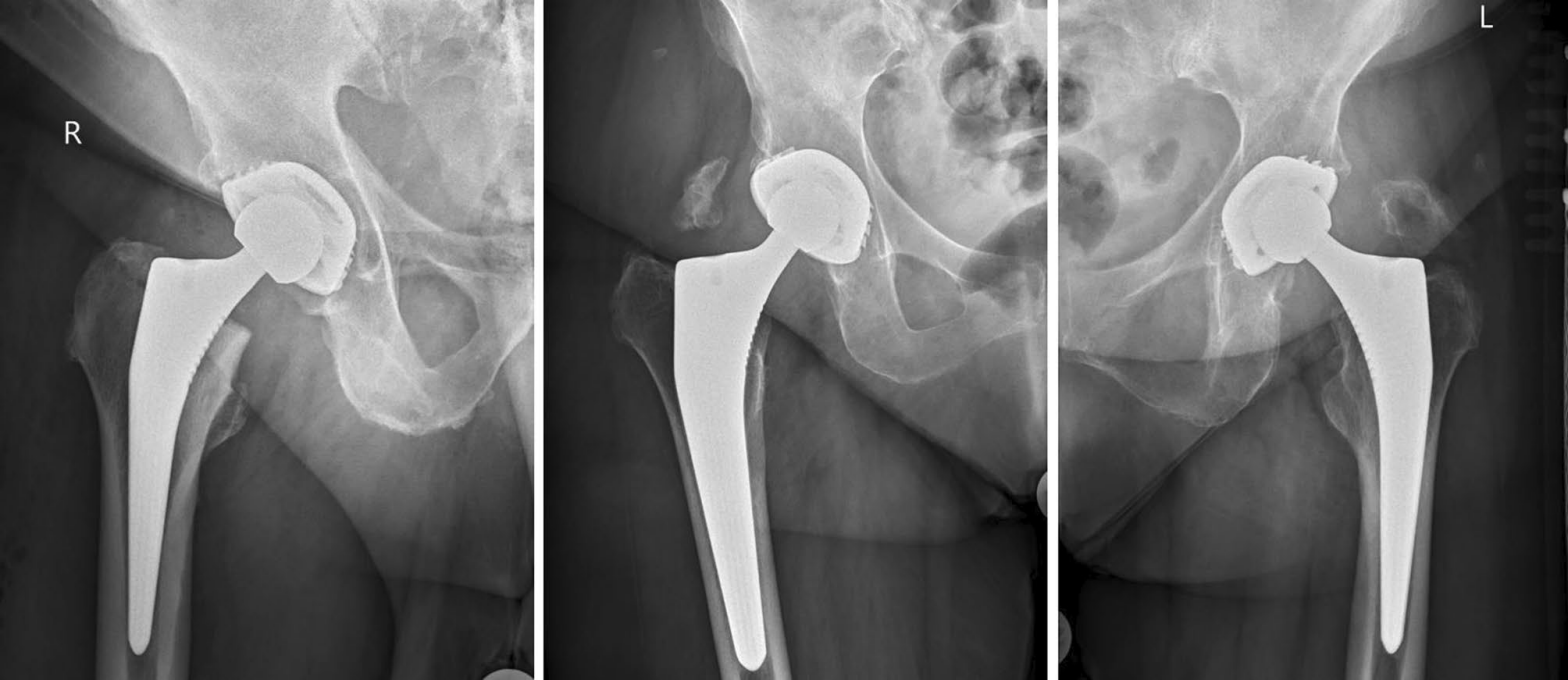
Evidence of HO Brooker grade I (left), II (central), and III (right).

All patients who underwent an anteroposterior radiography of the pelvis at a minimum of 12 months following THA were considered for inclusion. On admission, the age and sex of the patients were recorded, as were the causes of OA and the date of surgery. The degree of HO was assigned by a blinded assessor (trained radiology consultant) who was not involved in the clinical management of the patients. The modified Brooker Staging System was used to rate the efficacy of the interventions on anteroposterior radiographs (Table 1, Fig. 1). This classification differs from the original by an additional grade of 0, in which there is no sign of HO^42^.

**Table 1:** Modified brooker staging system.

Class	Radiographic findings
Brooker 0	No sign of heterotopic ossification
Brooker I	Bony islands in the soft tissue around the hip
Brooker II	Exophytes in the pelvis or proximal end of the femur with at least 1 cm between opposing bone surfaces
Brooker III	Exophytes in the pelvis or proximal end of the femur with less than 1 cm between opposing bone surfaces
Brooker IV	Bony ankylosis between proximal femur and pelvis

### Surgical technique and postoperative evaluation

All patients received a 1.5 g single shot of intravenous cefuroxime. All surgeries were performed by six senior surgeons using the Watson-Jones anterolateral approach^43^. The implant used for THA was the Smith & Nephew Polarstem (Smith & Nephew plc, Watford, England) cement-free, oxinium or ceramic femoral head, High-density crosslinked polyethylene (XLPE) inlay, cross-linked threaded acetabular cup. Anti-thrombotic prophylaxis with Rivaroxaban, 10 mg daily for six weeks, started 12 h after the index procedure. Patients were followed by a team of physiotherapists during hospitalization. Quadriceps strength exercise started on the first postoperative day. On the same day, patients mobilized weight bearing as tolerated using a forearm support frame. By the third postoperative day, patients progressed to mobilization using crutches. An outpatient rehabilitation program was set up and personalized for every patient. Patients were discharged on the sixth postoperative day.

### Statistical analysis

All statistical analyses were performed using the software IBM SPSS version 25. Continuous data were analysed using the mean difference (MD), while for dichotomic data, the odd ratio (OR) effect measures were calculated. The $\chi$^2^ test was performed to evaluate the rate of between groups HO. The analysis of variance (ANOVA) was performed to evaluate baseline comparability. Values of *P* < 0.05 were considered statistically significant.

### Ethical approval

This study was approved by the Ethics Committee of the Medical Faculty of the RWTH University of Aachen (project ID EK 438-20).

### Informed consent

All patients provided written consent to use their clinical and imaging data for research purposes.

## Results

### Recruitment process

**Figure 2:**
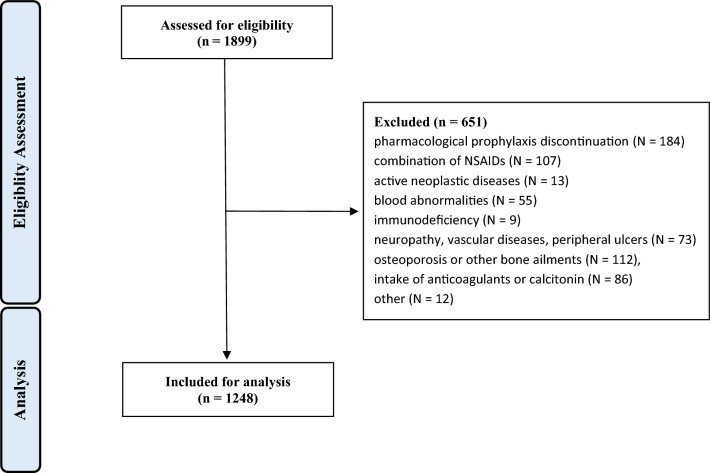
Diagram of the recruitment process.

Data from 1899 procedures were retrieved. 651 procedures were excluded with reason: pharmacological prophylaxis discontinuation (N = 184), combination of NSAIDs (N = 107), active neoplastic diseases (N = 13), blood abnormalities (N = 55), immunodeficiency (N = 9), severe peripheral neuropathy, vascular diseases, or presence of peripheral ulcers (N = 73), osteoporosis or other bone ailments which require stem and/or cup cementation (N = 112), concomitant intake of anticoagulants or calcitonin (N = 86), other (N = 12). This left 1248 eligible procedures for the present investigation (Fig. 2).

### Patient demographics

Data from 1248 patients were collected. 62% (767 of 1248 patients) were women. The mean age was 67.0 ± 2.9 years. The mean follow-up was 21.1 ± 10.8 months. At baseline, the ANOVA test evidenced no difference in sex, age and length of the follow-up (*P* > 0.1). The baseline demographic is shown in greater detail in Table 2.

**Table 2:** Demographic data of the patients (FU: follow-up).

Treatment arm	Procedures	Women	Mean age	Mean FU
Ibuprofen	619	60% (374 of 619)	64.3 ± 10.6	23.1 ± 10.6
Indomethacin	305	58% (177 of 305)	66.1 ± 11.8	21.9 ± 11.2
Control group	324	67% (216 of 324)	71.0 ± 9.1	18.4 ± 10.7

### Outcomes of interest

In the ibuprofen group, 73% (452 of 619) of patients evidenced Brooker 0, 17% (108 of 619) Brooker I, and 10% (59 of 619) Brooker II HO. In the indomethacin group, 77% (236 of 305) of patients evidenced Brooker 0, 16% (49 of 305) Brooker I, 6% (20 of 305) Brooker II. No patient in the ibuprofen and indomethacin group developed Brooker III or IV HO. In the control group, 64% (206 of 324) of patients evidenced Brooker 0, 21% (69 of 324) Brooker I, 3% (10 of 324) Brooker II, and 12% (39 of 324) Brooker III. No patient in the control group developed Brooker IV HO. These results are shown in greater detail in Table 3.

**Table 3:** Rate of HO according to the Brooker classification.

Treatment arm	Brooker 0	Brooker I	Brooker II	Brooker III	Brooker IV
Ibuprofen	73% (452 of 619)	17% (108 of 619)	10% (59 of 619)	0	0
Indomethacin	77% (236 of 305)	16% (49 of 305)	6% (20 of 305)	0	0
Control group	64% (206 of 324)	21% (69 of 324)	3% (10 of 324)	12% (39 of 324)	0

### Result syntheses

There was no evidence of a statistically significant difference in the rate of HO between indomethacin and ibuprofen at the last follow-up in all Brooker classes. On the other hand, both compounds demonstrated to be more effective than no pharmacological HO prophylaxis. These results are shown in greater detail in Table 4.

**Table 4:** Between-group comparison (HO: heterotopic ossification; OR: odd ratio).

Class of HO	Treatment arm	Ibuprofen	Indomethacin
Brooker 0	Indomethacin	OR 0.8; *P* = 0.1	
	Control group	OR 1.5; *P* = 0.002	OR 2.0; *P* = 0.0002
Brooker II	Indomethacin	OR 1.5; *P* = 0.1	
	Control group	OR 3.3; *P* = 0.0006	OR 2.2; *P* = 0.04
Brooker I	Indomethacin	OR 1.1; *P* = 0.6	
	Control group	OR 0.7; *P* = 0.2	OR 0.7; *P* = 0.1
Brooker III	Indomethacin	OR 0.5; *P* = 0.7	
	Control group	OR 0.006; *P* = 0.0003	OR 0.0; *P* = 0.002

## Discussion

According to the main findings of the present observational study, ibuprofen and indomethacin administered for three weeks were equally effective in the prophylaxis of HO following primary THA. Compared to the control group, both compounds were more effective in reducing the rate of HO occurrence following primary THA.

Several NSAIDs have been used as prophylaxis for HO following primary THA. However, despite several clinical studies, the most suitable compound is still debated. Indomethacin is effective in preventing HO following THA^31,35,44^. In the current literature, the effective dose of indomethacin varies from 75 to 150 mg daily, for one to 6 weeks^17,20,44–48^. Ibuprofen is also effective in preventing HO following THA^16,38,39^**,** ranging from 1200 to 1550 mg daily for 9–40 days^16,36–39^. We were unable to identify high-quality-controlled studies which compared ibuprofen versus indomethacin as prophylaxis for HO. A retrospective study included 200 THA patients compared three weeks of administration of indomethacin or ibuprofen, and a control group who did not receive any medication. Both treatment arms did not evidence the occurrence of moderate to severe HO at the last follow-up. In contrast, moderate or severe HO was found in patients who did not receive any prophylaxis. A recent level I of evidence Bayesian network meta-analysis, which included 6396 THAs, demonstrated that both ibuprofen and indomethacin were effective in the prevention of HO after THA^34^. The study compared Acetylsalicylic acid, Celecoxib, Diclofenac, Etoricoxib, Ibuprofen, Indomethacin, Meloxicam, Naproxen, Rofecoxib, and Tenoxicam at approximately one-year follow-up^34^. Among all compounds evaluated, ibuprofen and indomethacin demonstrated a modest capability to prevent HO^34^.

The prophylaxis of HO should be administered at the lowest dosages for the shortest possible period and associated with proton pump inhibitors (PPI) to prevent gastrointestinal complications. PPIs are generally considered safe and are often administered at high doses. The spectrum of side effects of PPI at high doses includes impaired absorption of nutrients and susceptibility to respiratory and gastrointestinal infections, kidney, liver, and cardiovascular disease, enteroendocrine tumours, and dementia^49–51^. In this respect, we remark that low and high doses of proton pump inhibitors have similar efficacy in preventing NSAIDs-induced mucus damage in patients without a history of gastrointestinal ailments^52–57^.

The institution where all THAs have been conducted has been accredited by “Endocert” since 2016 (EndoCert certificate, Centres of German Endoprosthetic, German Society for Orthopedics and Orthopedics). The EndoCert initiative represents the first certification system of medical centres for joint replacement in the world and was established in Germany in 2012. The EndoCert aims to maintain quality standards in primary and revision arthroplasty. The associated centres also develop and define standards as well as treatment processes, and they are subjected to continuous re-certification^58,59^. All surgeons obtained the certificate of senior operator of EndoCert, and were well beyond their learning curve, having each performed more than 500 hip arthroplasties.

The relatively small number of procedures included for analysis represents the most important limitations of the present study. Moreover, data was collected in a prospective fashion but analysed retrospectively, which represents an important source of selection bias during allocation concealment. Patients were not blinded, which increases the risk of performance bias. Additional confounding factors which may have influenced HO, such as comorbidity, age, sex, and concomitant pharmacological therapies, were not considered. The modified Brooker Staging System was used for assessment. The length of the follow-up of the present study was adequate. Although HO is already visible a few weeks postoperatively, its extent and Brooker grade cannot be definitively assessed until 12 months postoperatively^34^. Patients who developed an adverse reaction requiring suspension of the drug, or those who developed a pathology incompatible with the intake of ibuprofen or indomethacin within the first five postoperative days, were invited to discontinue the prophylaxis regimen and were included in the control group. The number of patients who changed to the control group for a perioperative discontinuation of NSAIDs was not recorded: this represents an important limitation of the present study. Brooker's classification was used to evaluate the efficacy of pharmacological compounds as prophylaxis for HO following THA. Other classifications are available to evaluate the extent of HO. However, the Brooker classification is the most commonly used and, therefore, the data presented in this article are comparable with other studies in this field. The Kellgren-Lawrence rating score system has been used to evaluate the severity of hip OA; Though this classification has been introduced to stage knee OA, it is valid and reliable also for hip OA^60–62^. The aetiopathogenesis of HO has been not fully clarified; however, it appears that the surgical approach may impact the rate of HO^63,64^. The posterior approach in THA seems to be associated with the lowest incidence of HO formation^65,66^, and the direct lateral approach has been reported to be at high risk for HO occurrence^67,68^. However, the current evidence on HO occurrence in different THA approaches has not been clarified and further investigations are required.

## Conclusion

A three-week administration of ibuprofen demonstrated similar efficacy as indomethacin in preventing HO following primary THA. The prophylaxis with ibuprofen or indomethacin was more effective in preventing HO compared to a control group of patients who did not receive pharmacological prophylaxis.

## Data Availability

The datasets generated and/or analysed during the current study are not publicly available but are available from the corresponding author on reasonable request.
